# The well-being valuation model: a method for monetizing the nonmarket good of individual well-being

**DOI:** 10.1007/s10742-016-0161-9

**Published:** 2016-10-25

**Authors:** James A. Sidney, Ashlin Jones, Carter Coberley, James E. Pope, Aaron Wells

**Affiliations:** Center for Health Research, Healthways, Inc., 701 Cool Springs Boulevard, Franklin, TN 37067 USA

**Keywords:** Health and well-being improvement, Health and well-being monetization, Exact matching, Medical cost minimization

## Abstract

The objective of this research is to advance the evaluation and monetization of well-being improvement programs, offered by population health management companies, by presenting a novel method that robustly monetizes the entirety of well-being improvement within a population. This was achieved by utilizing two employers’ well-being assessments with medical and pharmacy administrative claims (2010–2011) across a large national employer (*n* = 50,647) and regional employer (*n* = 6170) data sets. This retrospective study sought to monetize both direct and indirect value of well-being improvement across a population whose medical costs are covered by an employer, insurer, and/or government entity. Logistic regression models were employed to estimate disease incidence rates and input–output modelling was used to measure indirect effects of well-being improvement. These methodological components removed the burden of specifying an exhaustive number of regression models, which would be difficult in small populations. Members who improved their well-being were less likely to become diseased. This reduction saved, per avoided occurrence, US$3060 of total annual health care costs. Of the members who were diseased, improvement in well-being equated to annual savings of US$62 while non-diseased members saved US$26. The method established here demonstrates the linkage between improved well-being and improved outcomes while maintaining applicability in varying populations.

## Introduction

The objective of this research is to advance a novel method for evaluating and monetizing well-being improvement programs. While the scopes of these programs vary from program to program, their aim is to help facilitate behavior change toward healthier habits. As public and private organizations in addition to self-insured persons divert an increasing amount of resources toward well-being improvement programs, it is paramount that robust methods are utilized to properly evaluate the return on investment of these programs (Fidelity 2014). To date, several methods have been proposed for the purpose of monetizing health behaviors and health status improvement programs (Goetzel et al. 2009, 2005; Leutzinger et al. 2000; Kelly et al. 2010; Ozminkowski et al. 2004; Graham et al. 2007; Herman et al. 2006; Taitel et al. 2008). These methods are predicated on the hedonic price method in that the marginal implicit price of a health behavior, or the extent to which a program has modified behaviors, is reflected in the observed transactional costs for medical procedures and medications (Freeman III 1993). These methods, however, have only focused on the contribution of physical health risks to medical cost or specific outcomes in lieu of a total well-being effect. Furthermore, some current methods utilize independent data to derive trend coefficients without first administering matching procedures, such as propensity score matching or coarsened exact matching, prior to statistical analysis (Musich et al. 2015).

The assignment of a marginal implicit price to health behaviors based on an aggregate measure of health care cost is a necessary yet insufficient means by which to measure the efficacy of a well-being improvement program (Antweiler and Gulati 2015; Wang et al. 2015). It is a necessary step because at the present health and well-being risks are not billable events within an administrative claims file and hedonic methods are required to reveal the implicit price of these risks. A sufficient method would also incorporate the correlation well-being risks have with other risks and with the drivers of cost, namely, disease incidence, prevalence and severity (Mattke et al. 2009, 2013; Baicker et al. 2010). Moreover, the relationships between and among risks and cost should not be distilled to a series of linearly independent coefficients, as is the trademark of past research (Goetzel et al. 2005, 2013; Edington 2001). The valuation methodology presented here monetizes risk improvement from both implicit direct and indirect effects, which are captured through a collection of validated methods. Accordingly, we contend that current valuation methods undervalue well-being improvement programs by ignoring the interactive effects of risk mitigation and elimination.

Monetizing the direct and indirect value of well-being risks by their implicit effects on disease incidence and prevalence is important for two reasons. First, a disproportionate share of annual health care costs are borne by a relatively small proportion of the population and thus the implicit prices derived from hedonic methods are mostly measuring risk cost as a function of the minority characteristics (Cohen and Uberoi 2013). Second, among those with cost, more than 75 % of health care expenditures can be linked to individuals with chronic conditions such as diabetes, heart failure or chronic obstructive pulmonary disease (Anderson 2004). Current state valuation methods do not seek to quantify the monetary impact of well-being improvement attributed to onset and severity of such costly diseases and assume the impact is subsumed within an aggregate health care cost estimate.

Building upon existing research and applying quantitative methods from other disciplines, the method we propose can be utilized by program evaluators to monetize the comprehensive value of well-being improvement programs. This is achieved by examining a broad spectrum of well-being risks within an empirical framework consisting of a quasi-experimental technique, input–output model and logistic regression. The intent is that such a method will advance the measurement of what is currently unmeasured within traditional administrative health care cost accounting. From a much grander perspective, the goal would be to achieve validation of our nonmarket valuation methodology similar to the recommendations given to the National Oceanic and Atmospheric Administration for monetizing damage to environmental quality (Arrow et al. 1993).

## Data

Two distinct datasets were utilized in this study. One was drawn from a large national employer (n = 50,647) and used to quantify risk interactions over time. This large dataset provided the opportunity to capture robust variation within risk interactions. The second dataset was significantly smaller (n = 6170) and used to demonstrate applicability of the method to varying populations of size and risk profiles. Members from both datasets were included in the analysis if they completed two consecutive well-being assessments (WBA) between 2010 and 2011. Furthermore, both populations had a well-being improvement solution administered during 2011. The solution consisted of the WBA supplemented with interventions consisting of online education, telephonic outreach and onsite well-being coaches. The programs were offered to all members of the population but we evaluated adult members less than 65 during the study period. This was deliberate since members 65 and older were eligible for the Medicare program. This would create gaps in the data since Medicare-based medical claims would be unavailable for our study. Last, members were required to have at least 6 months of plan enrollment in each year.

The WBA captures information on more than fifty individual well-being risks across six domains: life evaluation, emotional health, physical health, healthy behaviors, work environment, and basic access (e.g. financial resources, community quality, health care access) (Evers et al. 2012). In addition to well-being risk data, the WBA provides demographic information, rates of worker absenteeism and presenteeism, and self-reported disease burden (Shi et al. 2013). To be eligible to participate in a well-being improvement programs, members were required to complete the WBA. Individual responses to WBA risk questions were categorized into binary indicators of whether a member was considered at risk. These binary indicators were then aggregated into factors based on previous research findings (Sears et al. 2014; Sidney et al. 2015). In total, eight significant factors emerged: Current Health Status, an indication of immediate health concerns; Health Maintenance, not meeting guidelines to maintain health; Mental Health, indication of risk for depression or anxiety; Negative Affect, indication of negative social or emotional feelings; Positive Affect, lack of positive social or emotional feelings; Financial Support, unable to afford basic needs; Financial Status, dissatisfaction with standard of living; and Strengths, expressed lack of daily purpose. In the last step of WBA data preparation, a member was considered at risk for a well-being category if any individual well-being risk within the category was at risk.

The outcome of interest, total health care costs, was derived from administrative claims data consisting of medical and pharmacy transactions. Costs were aggregated into a per person per year (PPPY) value for each reporting year based on 12 months of incurred claims with 3 months of runout. Additionally, based on administrative claims, binary variables were constructed to indicate whether members were diagnosed for the following diseases: diabetes, chronic obstructive pulmonary disease (COPD), asthma, coronary artery disease, and chronic heart failure. When inconsistent, objective claims-based indicators superseded the WBA self-reported disease indicators. Table 1 lists descriptive statistics for the population including demographics, health care costs and WBA-based risks.

**Table 1:** Descriptive Statistics for key factors in the Well-Being Valuation Method

Factor	Year 1 (n = 6170)		Year 2 (n = 6170)	
	Mean	STD	Mean	STD
Age	44.6	10.23	45.0	10.23
Gender (percentage female)	68.7 %	0.464	68.7 %	0.464
Eligible claims months	9.7	4.39	9.7	4.68
Per person per year total health care costs	US$2113.16	7592	US$2243.19	9583
Health maintenance risk	72.9 %	0.445	70.9 %	0.454
Current health status risk	57.2 %	0.495	58.3 %	0.493
Mental health risk	25.5 %	0.436	22.5 %	0.417
Negative affect risk	53.4 %	0.499	52.1 %	0.500
Positive affect risk	20.3 %	0.402	18.0 %	0.384
Financial status risk	23.0 %	0.421	20.7 %	0.405
Financial support risk	23.9 %	0.427	23.1 %	0.421
Strengths risk	17.1 %	0.377	15.8 %	0.365
Asthma prevalence	9.5 %	0.294	10.9 %	0.293
CAD prevalence	0.8 %	0.090	1.1 %	0.091
COPD prevalence	1.6 %	0.124	2.1 %	0.111
Diabetes prevalence	5.0 %	0.217	5.8 %	0.226
Heart failure prevalence	0.2 %	0.049	0.3 %	0.052
Non-diseased person prevalence	84.6 %	0.361	82.3 %	0.381
Newly diseased: asthma	–	–	1.4 %	0.118
Newly diseased: CAD	–	–	0.3 %	0.052
Newly diseased: COPD	–	–	0.5 %	0.072
Newly diseased: diabetes	–	–	0.8 %	0.090
Newly diseased: heart failure	–	–	0.1 %	0.028

## Methodology

The well-being valuation model (WBVM) is built upon several quantitative methods and is at the foundation a quasi-experimental method focused on computing the difference- between expected and actual trends of the study population. The relevant trends in this method apply to well-being risk factors, disease incidence, and disease prevalence. The trend specific differences are interpreted as the well-being improvement program’s effect and are monetized utilizing the total claim costs of the study population. The following subsections describe the way in which each quantitative method fits within the WBVM.

### Interactions and input–output models

The challenge in accurately valuing the entirety of all effects from risk change is indirect relationships risks demonstrate with one another. Elements of the input–output (IO) model methodology were integrated into the WBVM due to the mathematics and underlying theory supporting quantification of the full complement of interactive relationships or interdependencies between well-being risks, chronic conditions, and age (collectively referred to as inputs). The mathematical structure of the IO model explicitly accounts for the interdependencies, or correlations, between modeled factors and then non-parametrically quantifies the interaction across all inputs (Raa 2009). The resulting values, which are termed *final demand estimates*, reflect a set of input-specific estimates quantifying the one-time or sustained increase in population level risk attributed to not modifying prior risk levels.

The input–output model is typically used in regional economic analyses to model the impact of a change in the supply of one input on the output quantity of a good, and vice versa, and by extension, the impact to all inputs and goods within the regional economy. The impact results, or multipliers, are then often used to estimate the indirect benefits or costs of changes to the input and or output structure of the regional economy (Santos et al. 2013; Wiedmann 2009; Santos and Haimes 2004; Ritchie and Dowlatabadi 2014; Jewczak and Suchecka 2014). A recent study demonstrated an approach in which an input–output model was utilized to estimate the monetary impact to a region due to employee absenteeism at a local firm (Bankert et al. 2015). Other than this study and the current application, we are not aware of any other research that has integrated input–output modeling within well-being research to capture the interactive and marginal indirect effects of multiple risks over time on healthcare costs.

To calibrate application of the IO open model for well-being valuation, Spearman’s rho was calculated between the baseline and report year distributions of a given two-factor set. By using Spearman’s rho as the measure of correlation between factors over time we assumed a monotonic relationship between factor distributions (Hauke and Kossowski 2011; Spearman 1904). Calculated rho values were assumed to represent the fundamental physiological relationship between the factors and accordingly, be stable across time, customers and risk profiles. As an example of this methodological component, the Spearman’s rho between body mass index and life satisfaction level was 0.21; all other input to input correlations were measured in this manner. The Spearman’s rho values were aggregated into a 53 by 53 matrix and inverted in the statistical software R.

Specifying the correlation matrix involved quantifying the year-over-year trend in the average prevalence of each input. The input specific trend rates were used to form the vector of expected risks. The vector was comprised of the prevalence estimate for each dichotomous risk input and mean for all other inputs. For training, two versions of the vector were created; one based on the actual year-specific well-being risk distributions and the other based on expected risk trends. In terms of the latter vector, a zero inflated Poisson (ZIP) model was used to estimate the expected number of risks based on individual level baseline characteristics, including demographic, chronic condition and well-being risks as the covariates and actual follow-up values as the response. A trend was then computed for each well-being risk included in the correlation matrix as the average of individual-level risk trend values, which in turn were computed as the ZIP estimated value divided by the actual baseline value. Last, the derived trend estimate was multiplied by the actual average baseline risk value of the study population to form the expected follow-up risk level. All of these expected risk values constituted the final demand vector that was multiplied through the correlation matrix.

The multipliers were computed as the ratio of final demand estimates to the observed value of each input. Each multiplier quantified the percent contribution to the input trend due to the interaction among all of the modeled inputs. In other words, the multipliers represented the expected (1 year) trend in input risk (a) when the risk was left unchanged and (b) after accounting for the influence of all other modeled inputs and marginal indirect effects—externalities—therein. Alternatively, the multipliers can be interpreted as a ‘preventative’ effect since a preventative procedure, such as the flu shot, has both direct and indirect benefits. In the case of a flu shot, the direct benefit is a significantly reduced likelihood of contracting the flu; the indirect benefits include lower likelihood of developing other illnesses due to compromised immune system, higher quality of life during the flu, and decreased time out of work.

### Sources of value and effects for monetization

The monetary valuation of well-being change in terms of health care cost was determined from previously published literature and expected less actual costs from medical and pharmacy claims data (2010 and 2011). The expected costs were expressed in 2010 US dollars having utilized the medical Consumer Price Index from the Bureau of Labor Statistics. The effects of well-being change within WBVM are comprised of four sources: reduction in health care spend among the non-diseased, reduction in the likelihood of developing chronic disease, reduction in medical spend among newly diseased members and last, reduction in spend among those with disease.

#### Reduction in health care spend among the non-diseased

The marginal cost of being at risk for one or more of the well-being risks within a risk category was the basis for estimating savings due to the reduction of risks among the non-diseased. Studies have shown that people with well-being risks such as physical inactivity, smoking and obesity have higher utilization in terms of hospital stays, physician visits and prescription drugs (Pratt et al. 2000; Raebel et al. 2004; Harrison et al. 2012; Shi et al. 2012; White et al. 2013). Given the existence of these interactions and their manifestation in increased health care utilization, we chose to value reduction in the number and composition of well-being risks based on the differentially lower level of observed health care cost among those with no such risks or disease.

Two steps were followed to estimate the value of reduced risk, or alternatively, the additional cost associated with having one or more risks. In the first step, a ratio was computed of the PPPY medical expenditure for members with at least one risk within a risk category to the PPPY for members with no risks and no disease during the same time period. This ratio, or relative percent difference in cost between the two states (with risk/without risk), for each of the evaluated eight well-being risk factors is listed in Table 2.

**Table 2:** Relative increase in cost and expected growth rates of well-being risk factors

Well-being risk	Current health status	Mental health	Health maintenance	Positive affect	Negative affect	Financial support	Financial status	Strengths
Relative cost (%)^a^	44	10	16	20	11	55	4	15
Expected multiplier^b^	0.129	0.147	0.126	0.119	0.153	0.151	0.132	0.151

^a^Percent increase in cost for those with at least one risk within a well-being risk category, relative to members with no risks and disease conditions

^b^Correlation matrix multipliers for calculating the expected growth rate of the well-being risk category

The second step of the process for valuing risk reduction among the non-diseased was to monetize value in reducing the number of current risks and preventing development of additional risks. To estimate the number of risks reduced and avoided, there was a need to create an expected level of risks in the next measurement period that could be compared to the realized level of risks. The trend process described earlier concerning multipliers were applied to determine the difference between expected and actual number of risks. This difference was then multiplied by the marginal cost of having at least one risk in the category, aggregated over all members having the risk and then summed over all evaluated risk factors to derive cumulative savings within this component. Since members could have more than one category at risk, we specified a weighted average summation of risk category savings.

#### Reduction in disease incidence rate

The valuation of this savings component is based on evidence that a direct relationship exists between the composition and number of well-being risks, or more generally, well-being risk profile, and the probability of developing disease, and further, having disease increases the likelihood of developing co-occurring disease ( Wang et al. 2006). People that have chronic conditions spend significantly more in total health care expenditures than those without such conditions. In fact, it has been found that medical costs are approximately double for those with one chronic disease, compared to non-diseased persons, and costs increase exponentially as co-occurring diseases develop (Stanton 2006). Therefore, this method also incorporated savings attributed to a lower likelihood of chronic disease development and subsequent health care costs due to improved well-being risk profile.

Calculating the prevented risks required an estimate of the number of risks that would have been expected to occur in the next year absent intervention. This was achieved utilizing the same methodology detailed in Sect. 3.1. The expected prevalence by risk was then multiplied by risk-specific coefficients derived from logistic models estimating the impact of the expected change in risks on the likelihood of becoming newly diseased. Separate regression models were used to estimate the probability of developing each chronic disease including diabetes, coronary artery disease, chronic obstructive pulmonary disease, heart failure, and asthma. Independent variables in each model included demographic variables age and gender, chronic diseases excluding the dependent variable, and well-being risks aggregated to the factor level (see Table 3). Estimated coefficients from the models estimated on the training population were used to estimate change in the population’s likelihood of developing chronic disease based on changes in aggregate well-being risks by category, holding demographics and disease prevalence constant at their levels.

**Table 3:** Logistic model coefficients for the model estimating diabetes incidence

Factor	Beta	Wald statistic	*p* value	Odds ratios
Intercept	−11.51	19.155	<0.0001*	
Current health risk	1.28	9.405	0.002*	3.61
COPD (*1* = *yes*)	0.358	0.222	0.6369	1.43
Gender (1 = *female*)	−0.0002	0.0	0.9994	1.00
Age, *natural log*	1.40	4.270	0.039*	4.05
Asthma (*1* = *yes*)	0.227	0.285	0.594	2.89
Health maintenance risk	0.270	0.515	0.473	1.31
Support risk	0.771	6.188	0.013*	2.16

* Statistically significant at alpha = 0.05

An estimate of disease incidence avoided due to the reduction in the population level well-being risk profile was calculated as the product of the change in probability and number of non-diseased members within the population. A monetary value was then derived by multiplying the estimate of incidences avoided by the net baseline PPPY cost associated with having the disease; the net computation involved deducting the average annual cost of a diseased person from the average annual cost of a non-diseased person.

#### Reduction in cost of newly diseased

Although reduction in well-being risks reduces the likelihood of developing disease, all incidences will not be avoided. There is value, though, in terms of lowering well-being risk among those members who become newly diseased. This value was captured as the difference in health care cost among these members by differential risk severity level. Specifically, the percent cost difference among newly diseased members by disease and presence versus absence of a risk in a given factor was calculated. Savings were then computed as this difference multiplied by the average diseased PPPY and aggregated across all newly diseased members.

#### Reduction in cost among the diseased

The WBVM quantifies savings attributed to well-being risk reduction among diseased members using a similar methodology described in the previous section. Due to the low number of members eligible for this component of the savings analysis, a published estimate from the literature that utilized a matching-based methodology was chosen (Wells et al. 2012). The savings estimate from this study of US$24.67 per diseased member per month or US$294.07 PPPY (4.73 % savings rate) was applied to all actively engaged diseased members.

### Coarsened exact matching

A component of WBVM that is used in the instance where administrative claims are not available for a study population is Coarsened Exact Matching (CEM). CEM is a bias-reducing, quasi-experimental matching method employed to quantify a causal effect between cohorts (Iacus et al. 2009, 2011; Sidney et al. 2015). Studies have found CEM to yield causal estimates of a treatment with lower variance and bias of differing sample sizes relative to PSM (Wells et al. 2012; King et al. 2011). CEM was not used in the WBVM presented here but is an available component for the method in order to match the training and study populations to derive weights applicable to the study population.

## Results

The results presented below are observed from two consecutive years of WBA completion from the smaller employer population. The predicted values therein are calculated from observed 2 year data; out of sample predictions are beyond the intent of this study.

### Multiplier ratios and logistic coefficients

Based on the final demand estimates of the correlation matrix model, multipliers were calculated for each well-being factor (see Table 2). The multipliers ranged from 0.119 to 0.153, where lower values indicated less one-way, inter-temporal interdependence with other factors compared to larger values. As an example, the Current Health Status, Health Maintenance, and Positive Affect factors were less influenced by other factors while the Support and Negative Affect factors were more influenced by other risks.

Table 3 reports the logistic coefficients that were used to form empirical estimates of future disease incidence rates. Specifically focusing on the diabetes model, the Current Health Risk and Support Risk factors were significant predictors of the diabetes incidence rate, as well as the natural log of age; all other included covariates were not significant, a result likely attributed to the small sample size associated with diabetes incidence. Results of logistic models predicting incidence rate for the other evaluated conditions are available from the authors upon request.

### Monetization/sources of value

Examination of risk reduction within the non-diseased cohort and the expectation of future risks revealed that approximately 3200 separate well-being risks, across the eight factors, were either reduced or prevented through intervention. This reduction of risks is evidence of well-being improvement within the population; absent intervention and accounting for age, we would have expected risk increase. These prevented and reduced risks equated to PPPY savings of US$26.39. Table 4 demonstrates how the savings from one of the well-being factors, Current Health Risk, is determined. While the intervention’s efficacy was most prominent in the risk reduction cohort, the most impactful results, per person, were found in the delay of disease onset. The WBVM estimated 13 people to have avoided the onset of new disease within the following year, equating to a US$3059 PPPY savings. Those 13 people represent 7.3 % of the members who became newly diseased. Table 5 provides an example of how the savings value was found from delaying the onset of diabetes within the population. For the expected number of newly diseased members, reduction of well-being risks among the cohort saved US$49.88 PPPY. Reduction of risks among the already diseased resulted in savings of US$62.46 PPPY. Overall, the reduction of well-being risks resulted in cumulative savings of US$238,127 for the evaluated population, or US$38.59 PPPY. More detailed monetization results can be found in Table 6. While the return on investment is not reported for this study, the return is in line with comparable studies compiled by Baxter et al (2014).

**Table 4:** An example of valuing current health status risk

Observed members with risk: 2010	3527
Expected multiplier	0.129
Expected members with risk: 2011	3980
Observed members with risk: 2011	3597
Estimated members with avoided risk: 2011	383
Relative yearly cost of risk	US$787.93
Yearly cost savings due to risk reduction	US$301,415
Per person per year savings	US$48.85

Costs are expressed in 2010 US dollars

**Table 5:** Example of valuing the diabetes incidence rate reduction

2010 Disease profile		2011 Disease profile	
Prevalence rate	5.00 %	Incidence rate of newly-diseased	0.80 %
Population prevalence	309	Reduction in incidence rate from well-being change^a^	0.06 %
Mean cost of diabetics	$6178	Estimate of incidences avoided	3
Mean cost of non-diseased	$2002		
Cost of becoming diabetic	$4176	Estimated savings of avoided diabetic incidence	$12,588

Costs are expressed in 2010 US dollars

^a^Calculated from relevant logistic model

**Table 6:** Savings rate and total savings by source of value based on the well-being valuation method for the evaluated intervention program

Sources of value	Per person per year savings (SD)	Applied to relevant population^a^	Total
Intra year savings of all elements among non-diseased	US$26.39 (15.563)	5115	US$134,985
Incidence rate reduction savings	US$3058.98 (1919)	13	US$39,897
Savings on risk reduction among diseased	US$62.46 (4.970)	896	US$55,956
Savings on risk reduction among newly diseased	US$49.88 (1.768)	146	US$7289
Total savings			US$238,127
Total savings, per person per year			US$38.59

Costs are expressed in 2010 US dollars

* To prevent double-counting within the population, only one savings rate was computed for each individual

### Testing against regression

To evaluate comparability of the WBVM to another published valuation method, we followed an approach similar to Goetzel et al. (2009) and Leutzinger et al. (2000) to create the alternative. The alternative consisted of a series of cross-sectional, multivariate regression models estimated to predict the non-intervened health risk trend. Each model was specified with one of the eight health risks as the dependent variable and the remaining seven non-forecasted risks, in addition to age, gender and disease burden measured during the same time period as the right hand side. The estimated coefficients from each model were then used to predict the next year health risk by advancing the member’s age by 1 year. The individual level, forecasted next time period health risk estimates were then used in the cross-sectional model of health care expenditures. In this model, annual health costs of each member were regressed on the member’s demographics, disease burden, and forecasted health risk.

To determine the cost of an increased prevalence of a risk factor the difference of Year 1 mean costs and forecasted mean costs in Year 2 were calculated for those who were not at risk in Year 1 and were at risk in Year 2 for each factor. In the case of a decreased prevalence of a risk factor, the cost calculation remained consistent though it was calculated from those at risk in Year 1 and not at risk in Year 2. The relevant cost/savings of each risk factor and the expected change in prevalence can be found in Table 7. Of the eight risk factors, only Current Health Risk saw an expected growth in risk prevalence, equating to an annual increase of US$17,214 for the population. Despite the dissavings from that particular factor, the aggregate cost change across all factors resulted in total annual savings of approximately US$119,000 or US$19.28 PPPY.

**Table 7:** Monetization of value from regression analysis only

Risk factor	Cost of risk change per person per year^a^	Expected change in prevalence (%)	Total yearly costs (savings)
Current health risk	US$500.40	0.6	$ US$17,214
Mental health risk	US$207.68	−3.0	$ US$ (38,909)
Health maintenance risk	US$221.49	−1.7	$ US$ (23,036)
Positive affect risk	US$76.95	−2.3	$ US$ (10,890)
Negative affect risk	US$194.56	−1.3	$ US$ (15,133)
Financial support risk	US$132.90	−1.3	$ US$ (10,508)
Financial status risk	US$343.63	−1.8	$ US$ (38,253)
Strengths risk	US$ (26.05)	−0.4	$ US$579
Total costs (Savings)			$ US$ (118,935)
Per person per year costs (Savings)			$ US$ (19.28)

Costs are expressed in 2010 US dollars

^a^Costs are calculated as the difference of predicted costs of those at risk in Year 1 and not at risk in Year 2 (or vice versa)

It is important to note that the specified regression model cannot distinguish the different sources of well-being risk reduction value because the estimated coefficients are estimates of the marginal contribution of the risk to the outcome and are assumed to linearly independent. In the present application, the implication of these two limitations of linear regression modeling is that the well-being risk coefficients encompass all sources of value from risk reduction (e.g. risk reduction of non-diseased, risk reduction among diseased, and risk reduction of newly diseased). It is possible to include interaction covariates to quantify the different sources of value, however, this would require the inclusion of five diseases interacted with eight well-being factors across 2 years (80 new variables). The inclusion of interaction terms would greatly diminish model degrees of freedom and result in model over-specification.

## Discussion

The objective of this research was to develop a methodology that could be used to establish monetary estimates, and subsequently savings, associated with improvements in specific aspects of well-being over time. The WBVM was developed to accomplish this objective given that well-being is not a transacted good within the healthcare market. Well-being is an externality of observable healthcare expenditures tied to events such as hospitalizations, office visits and medication therapy (Harrison et al. 2012; Shi et al. 2012). The presence, absence and or interaction of observable types of healthcare expenditures across a population in conjunction with measured well-being allows for estimation of the unobservable, yet related attributes expressed in individual well-being. The WBVM is a novel approach combining a series of sophisticated methodologies that in totality yield a monetary value for these attributes. In the study population, the value of well-being improvement totaled US$38.59 PPPY or approximately twice the gross savings estimated by current valuation methods.

The monetization of a non-monetary good requires first identifying the good to be valued followed by application of state-of-the art methods to quantify a dollar value and then ending with a comparison to other valuation methods applied to the same goods. This sequence of steps is required when valuing specific elements of well-being, such as the social element, due to the nonexistence of readily available, observable market transaction information concerning the good. In the present study, the first step involved identification of the specific elements of well-being to value; choosing to evaluate more than fifty well-being risks assessed within the WBA and then to aggregate specific risks uniquely into eight factors based on factor analysis and heuristics. The focus on a wide spectrum of well-being risks is an advancement compared to existing methodologies that rely primarily reliance on physical and mental health aligned risks (Burton et al. 2003; Goetzel et al. 2012; Yen et al. 2003). Moreover, this study monetized multiple sources of value in relation to outcomes concerning employee health care costs. Specifically, four valuation components of risk reduction within the non-diseased cohort, risk reduction among diseased members, risk reduction within the newly diseased and reduced disease incidence were identified for the purpose of more clearly demonstrating the impact and linkage between improved well-being and improved outcomes.

The second step involved application of state-of-the art methods including a series of multivariate regression models, elements of input–output models, and Coarsened Exact Matching; to our knowledge, the WBVM is the first to combine all of these advanced methodological techniques to value non-market health care goods, namely well-being. Current methods for valuing well-being generally fall into one of two classes—nonexperimental and causal. Nonexperimental studies are numerous and principally rely on the seminal work of Goetzel et al. (2005), with examples including (Bowen et al. 2009; Kelly et al. 2010). The methodological limitations of these studies include use of cross-sectional health risk values for change analysis, linear independent cost estimation via multivariate regression modeling, and external cost values applied without matching to a study population. These studies have also typically evaluated a series of health risks whereas other nonexperimental studies have valued individual health risks such as smoking (Solberg et al. 2006), obesity (Baker et al. 2008) and physical activity (Wu et al. 2011). The limitation of these approaches includes the above as well as lack of control for other moderating health risks on the outcome. In terms of methods seeking causality, at least one randomized control study and several quasi-experimental studies using either CEM or Propensity Score Matching have been published (Sidney et al. 2015; Meenan et al. 2010; White et al. 2013).

A fundamental advantage of WBVM relative to these current methods is the correlation matrix model to derive an estimate of risk trend absent intervention. For current methodologies to achieve inter-temporal interactions among well-being risks, similar to the input–output model, more than fifty regression models each specified with all risks and demographic parameters would need to be estimated. This type of approach is unnecessarily cumbersome and subject to bias stemming from multiple hypothesis testing and endogeneity given the correlation that exists between risks and modeled outcomes. Furthermore, when this approach was attempted by the study, it did not capture the full extent of value of risk reduction.

### Limitations

While the WBVM advances the state-of-the art for valuing well-being, the approach is not without limitations. First and foremost, more research is needed across longer time horizons and more diverse populations in order to more accurately derive the expected outcome trend and understand variance between actual and expected trends. The longer time horizon would allow validation of the accuracy and robustness of predicted values over time. Second, the WBVM is complex and may be challenging to immediately understand; this concern is buffered by the scientific basis of the method. Additionally, use of an external data source for the matched trend component may not be preferred by some researchers due to preference for use of their own information. As a potential solution, the method could be applied based solely on the study population given that there are two or more distinct time periods of data, sufficient sample size in both treated and comparison groups, and a comprehensive population health improvement strategy (as opposed to a specific program) has been implemented. Last, this method does not establish causality between well-being improvement and the intervention program. Instead, it measures population-level well-being change that incorporates multiple aspects of a well-being improvement strategy.

## Conclusion

The objective of this research was to develop a methodology that could be used to establish monetary estimates, and subsequently savings, associated with well-being improvement over time. This research expands valuation of well-being beyond physical and mental health risks to encompass all aspects of total population well-being. To achieve this, a set of peer-reviewed methods were combined within a single valuation framework that accounted for multiple interactions between a wide array of well-being risks known to impact employee health care costs. The method established here empirically demonstrates the linkage between improved well-being and improved outcomes and yielded an estimate of gross savings equal to approximately US$40 PPPY, nearly twice the amount found with current methods.
